# Association of day 4 cumulative fluid balance with mortality in critically ill patients with influenza: A multicenter retrospective cohort study in Taiwan

**DOI:** 10.1371/journal.pone.0190952

**Published:** 2018-01-09

**Authors:** Wen-Cheng Chao, Chien-Hua Tseng, Ying-Chun Chien, Chau-Chyun Sheu, Ming-Ju Tsai, Wen-Feng Fang, Yu-Mu Chen, Kuo-Chin Kao, Han-Chung Hu, Wann-Cherng Perng, Kuang-Yao Yang, Wei-Chih Chen, Shinn-Jye Liang, Chieh-Liang Wu, Hao-Chien Wang, Ming-Cheng Chan, TSIRC (Taiwan Severe Influenza Research Consortium)

**Affiliations:** 1 Division of Chest Medicine, Department of Internal Medicine, Taichung Veterans General Hospital, Taichung, Taiwan; 2 Department of Medical Research, Taichung Veterans General Hospital, Taichung, Taiwan; 3 Department of Business Administration, National Changhua University of Education, Changhua, Taiwan; 4 Department of Critical Care Medicine, Taichung Veterans General Hospital, Taichung, Taiwan; 5 Institute of Epidemiology and Preventive Medicine, National Taiwan University, Taipei, Taiwan; 6 Division of Chest Medicine, Department of Internal Medicine, National Taiwan University Hospital, Taipei, Taiwan; 7 Division of Pulmonary and Critical Care Medicine, Kaohsiung Medical University Hospital, Kaohsiung, Taiwan; 8 School of Medicine, College of Medicine, Kaohsiung Medical University, Kaohsiung, Taiwan; 9 Division of Pulmonary and Critical Care Medicine, Department of Internal Medicine, Kaohsiung Chang Gung Memorial Hospital, Kaohsiung, Taiwan; 10 Department of Respiratory Care, Chang Gung University of Science and Technology, Chiayi, Taiwan; 11 Department of Thoracic Medicine, Chang Gung Memorial Hospital, Taoyuan, Taiwan; 12 Department of Respiratory Therapy, Chang-Gung University College of Medicine, Taoyuan, Taiwan; 13 Division of Pulmonary and Critical Care Medicine, Department of Internal Medicine, Tri-Service General Hospital, National Defense Medical Center, Taipei, Taiwan; 14 Department of Chest Medicine, Taipei Veterans General Hospital, Taipei, Taiwan; 15 Institute of Emergency and Critical Care Medicine, School of Medicine, National Yang-Ming University, Taipei, Taiwan; 16 Division of Pulmonary and Critical Care, Department of Internal Medicine, China Medical University Hospital, Taichung, Taiwan; 17 Center for Quality Management, Taichung Veterans General Hospital, Taichung, Taiwan; 18 Office of Medical Administration, Taichung Veterans General Hospital, Taichung, Taiwan; 19 Central Taiwan University of Science and Technology, Taichung, Taiwan; Azienda Ospedaliero Universitaria Careggi, ITALY

## Abstract

**Background:**

Fluid balance is a fundamental management of patients with sepsis, and this study aimed to investigate the impact of cumulative fluid balance on critically ill patients with influenza admitted to an intensive care unit (ICU).

**Methods:**

This multicenter retrospective cohort study was conducted by the Taiwan Severe Influenza Research Consortium (TSIRC) which includes eight medical centers. Patients with virology-proven influenza infection admitted to ICUs between October 2015 and March 2016 were included for analysis.

**Results:**

A total of 296 patients were enrolled (mean age: 61.4±15.6 years; 62.8% men), and 92.2% (273/296) of them required mechanical ventilation. In the survivors, the daily fluid balance was positive from day 1 to day 3, and then gradually became negative from day 4 to day 7, whereas daily fluid balance was continuously positive in the non-survivors. Using the cumulative fluid balance from day 1–4 as a cut-off point, we found that a negative cumulative day 1–4 fluid balance was associated with a lower 30-day mortality rate (log-rank test, *P =* 0.003). To evaluate the impact of shock on this association, we divided the patients into shock and non-shock groups. The positive correlation between negative day 1–4 fluid balance and mortality was significant in the non-shock group (log-rank test, *P =* 0.008), but not in the shock group (log-rank test, *P =* 0.396). In a multivariate Cox proportional hazard regression model adjusted for age, sex, cerebrovascular disease, and PaO2/FiO2, day 1–4 fluid balance was independently associated with a higher 30-day mortality rate (aHR 1.088, 95% CI: 1.007–1.174).

**Conclusions:**

A negative day 1–4 cumulative fluid balance was associated with a lower mortality rate in critically ill patients with influenza. Our findings indicate the critical role of conservative fluid strategy in the management of patients with complicated influenza.

## Introduction

Influenza infection was estimated to affect nearly five million people worldwide and cause approximately 250,000 to 500,000 deaths in 2014 [1]. Taiwan experienced an influenza epidemic, predominantly type A (H1N1), in the spring of 2016, and the Taiwan Severe Influenza Research Consortium (TSIRC) was established to investigate management strategies including fluid balance for patients admitted to an intensive care unit (ICU) for complicated influenza.

Prompt and adequate fluid administration to restore the microcirculation is a fundamental management strategy for patients with sepsis [2, 3], and increasing evidence has suggested an association between worse outcomes and positive cumulative fluid balance in patients with sepsis and acute respiratory distress syndrome (ARDS) [4–6]. Optimal fluid administration is a complex process in the management of sepsis, and inadequate early fluid rescue may lead to endothelial damage and additional difficulties in subsequent fluid administration strategies [7]. Sepsis has been reported to be a dysregulated host-pathogen response [8], indicating the potential need for pathogen-specific fluid management strategies. Thus, optimal fluid therapy in patients with sepsis depends on both the status of sepsis and the responsible pathogens, and must be tailored to the clinical condition of each patient [9].

Influenza virus primarily binds with sialic-acid-containing surface receptors in epithelial cells, most frequently in the airway, including the lung, through hemagglutinin. Therefore, severe complicated influenza infection generally presents with severe respiratory distress, including ARDS, rather than profound shock [10, 11]. This unique feature of primary lung damage allows for the investigation of the impact of cumulative fluid balance in critically ill patients with influenza who present with severe respiratory distress. In this multicenter study, we enrolled patients admitted to ICUs for complicated influenza to investigate the impact of cumulative fluid status on 30-day mortality rate.

## Methods

### Study design

In this multicenter retrospective cohort study, we enrolled patients admitted to the ICUs at eight participating hospitals in Taiwan, including four hospitals in northern Taiwan, two hospitals in central Taiwan, and two hospitals in southern Taiwan. The study was approved by the Institutional Review Boards of the eight participating hospitals (Taichung Veterans General Hospital CE16093A, National Taiwan University Hospital 201605036RIND, Taipei Veterans General Hospital 2016-05-020CC, Tri-Service General Hospital 1-105-05-086, Chang-Gung Memorial Hospital 201600988B0, China Medical University Hospital 105-REC2-053(FR), Kaohsiung Medical University Hospital KUMHIRB-E(I)-20170097, Kaohsiung Chang-Gung Memorial Hospital 201600988B0). Written informed consent was waived due to minimal risk, and because all patient information was anonymized and de-identified before analysis.

We screened all patients admitted to the ICUs of the eight hospitals between October 1, 2015 and March 31, 2016 with a diagnosis of virology-proven influenza confirmed by the Taiwan Centers for Disease Control (CDC) based on the rapid influenza diagnostic test (RIDT), reverse transcription-polymerase chain reaction (RT-PCR), or viral culture. Patients without complete fluid data during the initial 3 days including those without pre-ICU admission fluid data, and those who died within 3 days post-intubation were excluded from analysis. The grading of ARDS in this study was in accordance with the Berlin definition [12].

### Measurements

Data were collected using a standardized case report form at the eight hospitals.

Medical records were reviewed to obtain data on total daily input and output, and relevant characteristics including demographics, comorbidities, initial laboratory tests including serum C-reactive protein level, subtypes of influenza, severity score (Acute Physiology and Chronic Health Evaluation II (APACHE II)). In addition, we also assessed the use and duration of vasopressors.

### Statistical analysis

Data were presented as frequencies (percentages) for categorical variables and as means ± standard deviations for continuous variables. Differences between the survivor and non-survivor groups were analyzed using the Student’s *t*-test for continuous variables and Fisher’s exact test for categorical variables. Kaplan-Meier analysis was used to test the association between 30-day mortality and cumulative day 1–4 fluid balance status. A Cox proportional hazards regression model was constructed to identify independent variables that predicted 30-day mortality. Given that serum lactate is highly correlated with the use of vasopressor, we included the use of vasopressor in the Cox regression model in this study. Statistical significance was set at a two-sided *P* value of less than 0.05. All data were analyzed using SPSS software version 22.0 (SPSS Inc., Chicago, IL, USA).

## Results

### Demographic and relevant data

**Fig 1:**
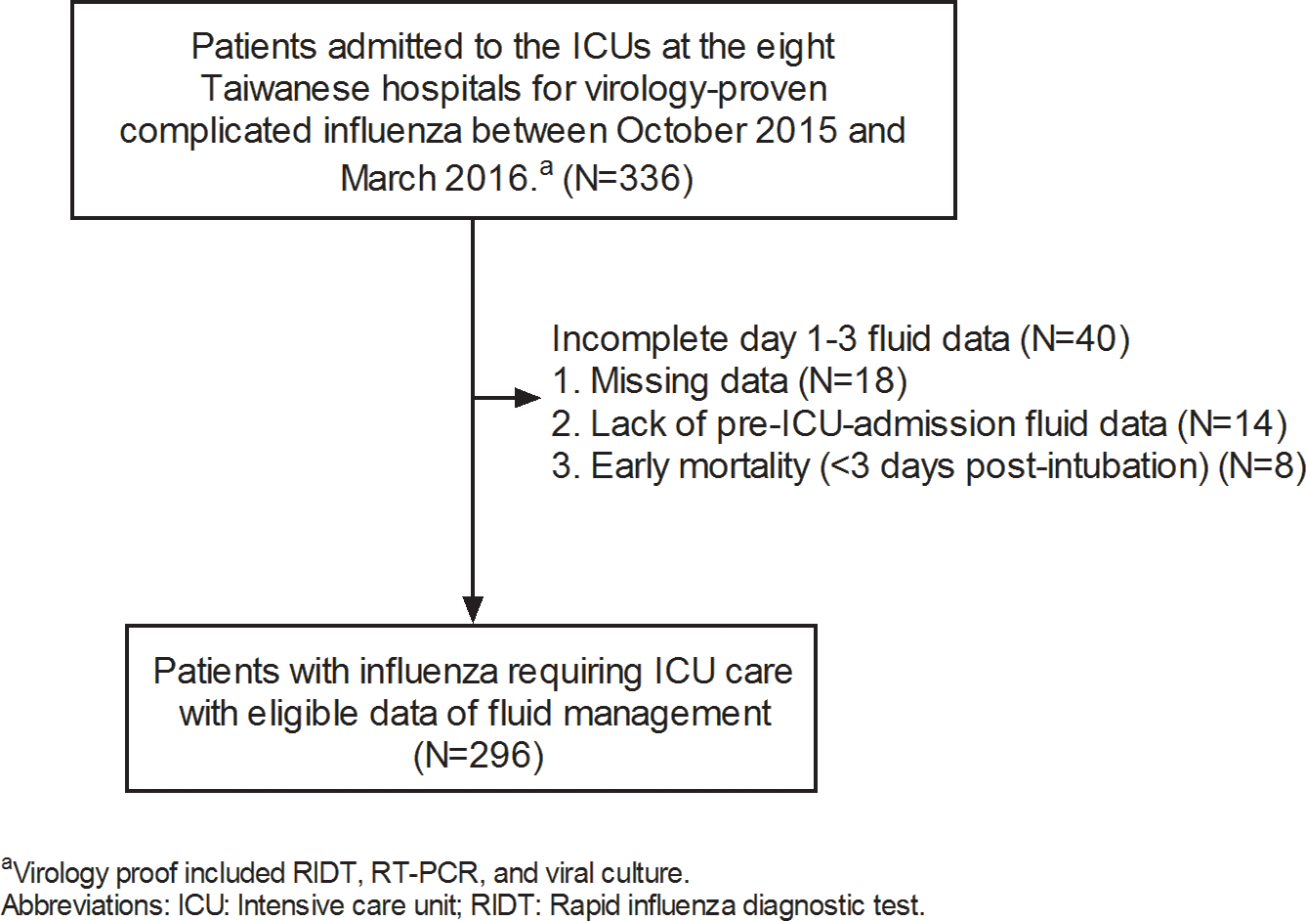
Flow chart of patient enrollment.

A total of 336 patients admitted to ICUs at the participating teaching hospitals for virology-proven influenza between October 2015 and March 2016 were enrolled. Of these 336 patients, 18 were excluded due to missing data, and 14 with late admission to the ICU were also excluded because of a lack of initial fluid data. As the focus of this study was to investigate cumulative fluid status, we also excluded eight subjects who died within 3 days post-intubation. The remaining 296 patients were eligible for analysis (Fig 1).

Table 1 summarizes the demographic and other relevant data. The mean age was 61.4 ± 15.6 years, and 62.8% were men (see S1 Dataset for details). The most common underlying comorbidities were type II diabetes mellitus (30.7%) and malignancy (14.9%). To investigate the factors associated with mortality, we categorized the 296 subjects into survivor and non-survivor groups according to mortality at 30 days. Compared with the survivor group, those in the non-survivor group were more likely to have a history of cerebrovascular disease (16.4% vs. 7.5%, *P =* 0.06). There were no significant differences in other relevant variables including clinical manifestations. In addition, the distribution of influenza types was also similar between the two groups. Consistent with data reported by the Taiwan CDC [13], type A influenza (75.7%, 224/296) was the most common type of influenza.

**Table 1:** Characteristics of the 296 patients stratified by 30-day mortality.

	All	Survivors	Non-survivors	*P* value
	(N = 296)	(N = 241)	(N = 55)	
**Basic data**				
Age (years)	61.4±15.6	60.8±15.5	64.1±16.2	0.15
Male %	186 (62.8%)	150 (62.2%)	36 (65.5%)	0.76
Body weight (kg)	67.1±14.8	67.3±15.2	66.1±12.9	0.58
Malignancy	44 (14.9%)	34 (14.1%)	10 (18.2%)	0.53
Type II diabetes mellitus	91 (30.7%)	72 (29.9%)	19 (34.5%)	0.52
Cerebrovascular disease	27 (9.1%)	18 (7.5%)	9 (16.4%)	0.06
Chronic airway disease	29 (9.8%)	24 (10%)	5 (9.1%)	>0.99
End-stage renal disease	19 (6.4%)	16 (6.6%)	3 (5.5%)	>0.99
Congestive heart failure	32 (10.8%)	25 (10.4%)	7 (12.7%)	0.63
**Major complication of influenza**				
Pulmonary abnormalities	290 (98.0%)	235 (97.5%)	55 (100%)	0.60
Neurological abnormalities (seizure)	6 (2.0%)	4 (1.7%)	2 (3.6%)	0.31
Cardiac abnormalities (myocarditis)	12 (4.1%)	8 (3.3%)	4 (7.3%)	0.25
**Subtypes of influenza**				
Type A	224 (75.7%)	185 (76.8%)	39 (70.9%)	0.37
Type B	24 (8.1%)	17 (7.1%)	7 (12.7%)	
Positive, unknown subtype	48 (16.2%)	39 (16.2%)	9 (16.4%)	
**Laboratory data and severity**				
White blood cell count (count/μl)	10329.3±6076.1	9984.3±5578.3	11834.5±7774.4	0.10
Hemoglobin (g/dL)	11.8±2.7	11.9±2.7	11.6±2.8	0.55
Platelet (10^3^/μL)	160.3±89.6	163.2±90.5	147.9±85.3	0.25
Albumin (mg/dL)	2.9±0.6	2.9±0.5	2.7±0.7	0.11
Creatinine (mg/dL)	1.7±1.9	1.6±1.7	2.4±2.8	0.07
C-reactive protein (mg/dL)	15±10.1	14.6±9.9	16.8±10.7	0.17
Lactate (mg/dL)	26.3±31.2	22.5±22.5	42.1±51.0	<0.01
APACHE II	22.2±8.4	21.2±8.2	26.7±8.1	<0.01
PaO2/FiO2	129.3±96.3	136.5±101.3	98.3±62.9	<0.01
**Management and outcomes**				
Intubation (%)	273 (92.2%)	222 (92.1%)	51 (92.7%)	>0.99
non-ARDS (%)	20 (7.4%)	19 (8.6%)	1 (2.0%)	0.09
mild ARDS (%)	28 (10.3%)	26 (11.8%)	2 (3.9%)	
moderate ARDS (%)	79 (29.0%)	64 (29.0%)	15 (29.4%)	
severe ARDS (%)	145 (53.3%)	112 (50.7%)	33 (64.7%)	
Receiving hemodialysis (%)	50 (16.9%)	33 (13.7%)	17 (30.9%)	<0.01
Vasopressor use (%)	145 (49%)	107 (44.4%)	38 (69.1%)	<0.01
Vasopressor (days)	6.6 ± 7.1	6.4±7.6	7.2±5.6	0.57
Ventilation (days)	17.4±13.8	17.8±14.7	14.8±6.8	0.06
ICU stay (days)	19.1±17.2	20.4±18.4	12.8±6.7	<0.01
Hospital stay (days)	32.8±24.5	36.9±25.2	14.8±6.8	<0.01

Data are presented as mean ± SD and N(%). ICU: Intensive Care Unit; APACHE II: Acute Physiology and Chronic Health Evaluation; ARDS: Acute Respiratory Distress Syndrome; ECMO: Extracorporeal Membrane Oxygenation.

Given that the eight participating hospitals were all teaching hospital, the enrolled patients had severe complicated influenza, with a high APACHE II score (22.2±8.4), a low PaO2/FiO2 ratio (129.3±96.3), and a 92.2% (273/296) intubation rate. Moreover, of the 273 intubated patients, 92.3% (252/273) had ARDS, 23.4% (64/273) received prone-ventilation, and 15.4% (42/273) received extracorporeal membrane oxygenation (ECMO) during ICU admission. Importantly, even though the severity of illness was quite high, only 49% (145/296) of the patients had shock defined by the use of any vasopressors during the ICU admission. Collectively, these data demonstrated a high rate of severe oxygenation failure with a relatively low rate of circulatory shock in the enrolled patients with complicated influenza.

### Daily and cumulative fluid status

Table 2 details the daily and cumulative input (I), output (O), and fluid balance (I-O) data. Given that the enrolled patients all had virology-proven influenza, the fluid administration was relatively modest, with 2094±1726 mL on day 1, 2649±1145 mL on day 2, and 2551±1141 on day 3. A slightly more positive fluid balance was found in the non-survivor group compared with the survivor group, with day 1 fluid balance 1076±1946 vs. 593±1691 mL (*P =* 0.069), day 2 fluid balance 1280±1439 vs. 598±1285 mL (*P =* 0.001), and day 3 fluid balance 613±1667 vs. 279±1235 mL (*P =* 0.093). A negative fluid balance was noted in the survivor group from day 4 (day 4: -47±1128, day 5: -227±1296, day 6: -452±2004, and day 7: -202±1148 mL), whereas a continuous positive fluid balance was noted in the non-survivor group (day 4: 704±1570, day 5: 93±1625, day 6: 453±1310, and day 7: 468±1516 mL). Therefore, compared with those in the non-survivor group, the patients in the survivor group had a less positive fluid balance on day 1–3 and a negative fluid balance from day 4. The cumulative data further demonstrated distinct differences in fluid balance status between the survivor and non-survivor groups, with a strong statistically significant difference in cumulative day 1–4 fluid balance (1384±3159 vs. 3635±4303 mL, *P*<0.001), and these differences remained robust in cumulative day 1–5, day 1–6, and day 1–7 fluid balance. Taken together, these findings showed distinct differences in fluid balance status with a less positive fluid balance in the survivor group.

**Table 2:** Daily and cumulative fluid status of the 296 patients with influenza by 30-day mortality.

	All (N = 296)			Survivors (N = 241)			Non-survivors (N = 55)			*P* value^a^
	Input (I)	Output (O)	IO balance	Input (I)	Output (O)	IO balance	Input (I)	Output (O)	IO balance	
**Daily fluid status**										
Day 1	2094±1726	1408±1027	684±1748	2038±1666	1433±1003	593±1691	2328±1959	1300±1132	1076±1946	0.069
Day 2	2649±1145	1936±1186	727±1340	2581±1110	2000±1196	598±1285	2942±1256	1662±1110	1280±1439	0.001
Day 3	2551±1141	2232±1441	341±1329	2487±983	2226±1211	279±1235	2828±1650	2257±2203	613±1667	0.093
Day 4	2444±935	2367±1286	93±1254	2409±914	2467±1246	-47±1128	2595±1014	1925±1376	704±1570	0.001
Day 5	2395±925	2574±1362	-170±1363	2366±881	2604±1322	-227±1296	2531±1105	2437±1543	93±1625	0.128
Day 6	2345±854	2646±1896	-301±1934	2293±755	2745±1973	-452±2004	2601±1213	2148±1364	453±1310	0.003
Day 7	2402±902	2505±1273	-94±1236	2329±826	2541±1219	-202±1148	2782±1164	2314±1524	468±1516	0.008
**Cumulative fluid status**										
Day 1–2	4605±2431	3223±1889	1382±2422	4471±2306	3305±1862	1166±2268	5185±2862	2867±1979	2318±2835	0.001
Day 1–3	7109±3172	5400±2861	1710±3011	6903±2937	5472±2725	1431±2807	8013±3949	5083±3404	2930±3557	0.001
Day 1–4	9545±3709	7743±3686	1802±3504	9302±3465	7918±3538	1384±3159	10608±4512	6974±4227	3635±4303	<0.001
Day 1–5	11875±4295	10239±4460	1637±3968	11629±4077	10468±4317	1161±3615	12955±5047	9234±4955	3721±4742	<0.001
Day 1–6	14109±4681	12759±5248	1349±4553	13865±4412	13145±5054	720±4207	15178±5636	11070±5778	4108±5009	<0.001
Day 1–7	16194±4789	15027±5899	1249±4850	15905±4307	15518±5558	510±4429	17478±6406	12879±6856	4474±5313	<0.001

^a^comparison of IO balance between the survivors and non-survivors. Data are presented as mean ± SD.

### Cumulative fluid balance day 1–4 was associated with 30-day mortality

**Fig 2:**
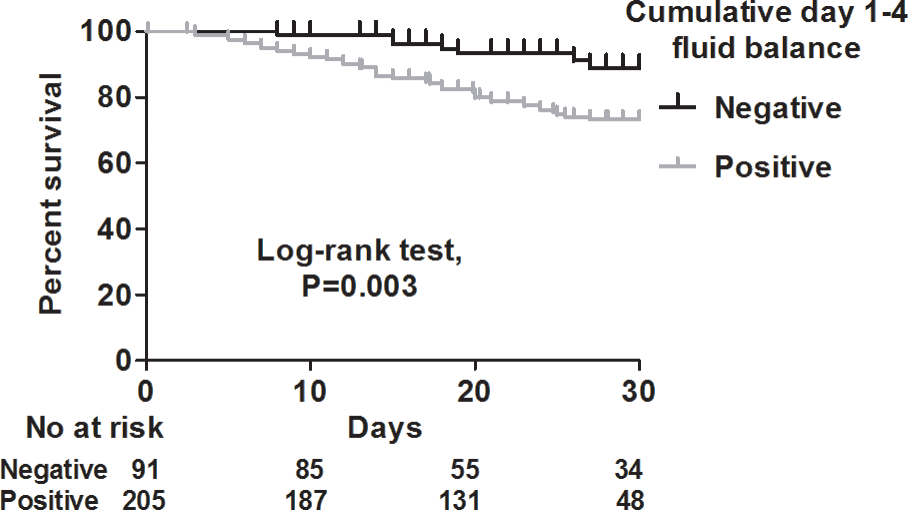
Kaplan-Meier survival curves for positive (gray) and negative (black) cumulative day 1–4 fluid balance.

**Fig 3:**
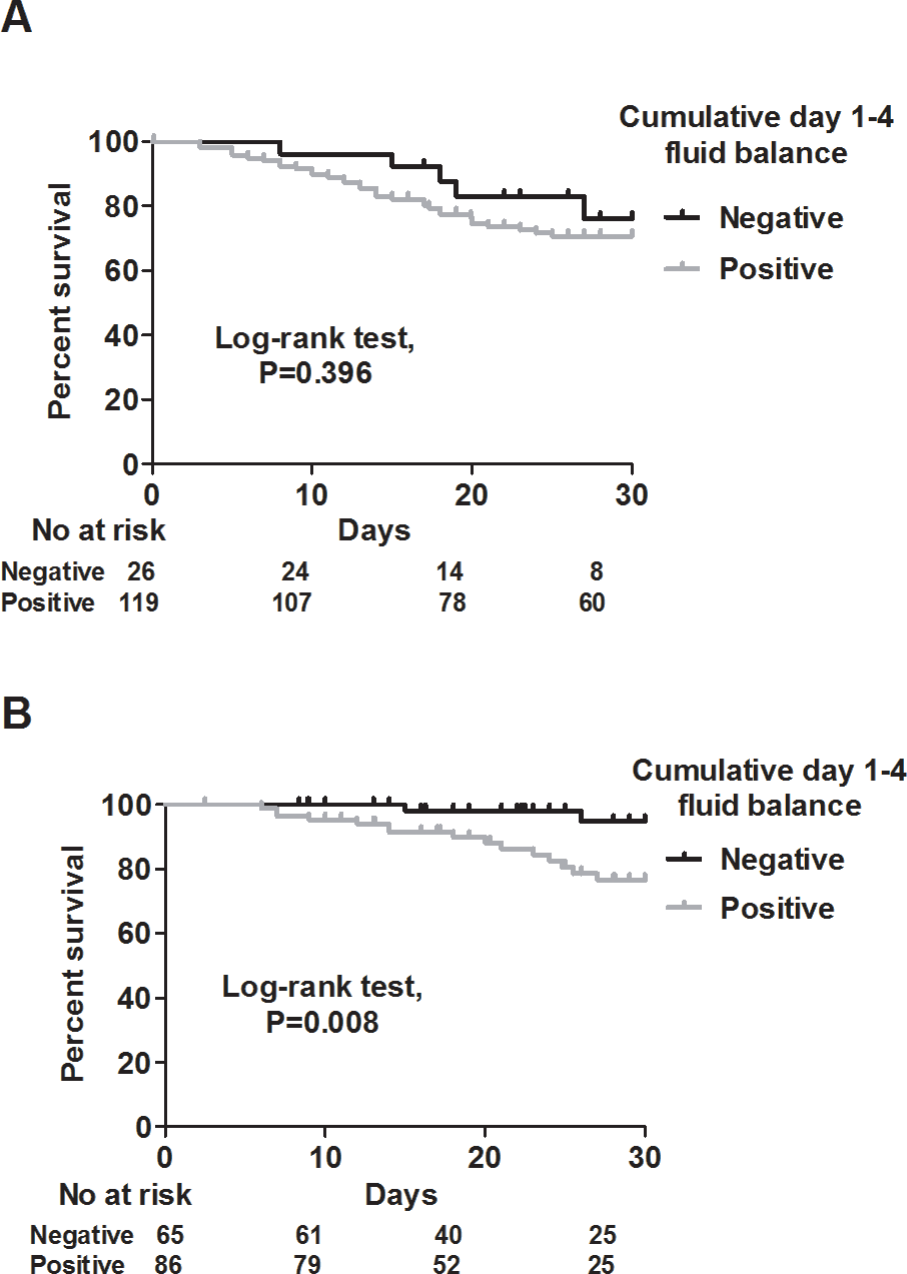
Kaplan-Meier survival curves for positive (gray) and negative (black) cumulative day 1–4 fluid balance in the shock (A) and non-shock (B) groups.

**Fig 4:**
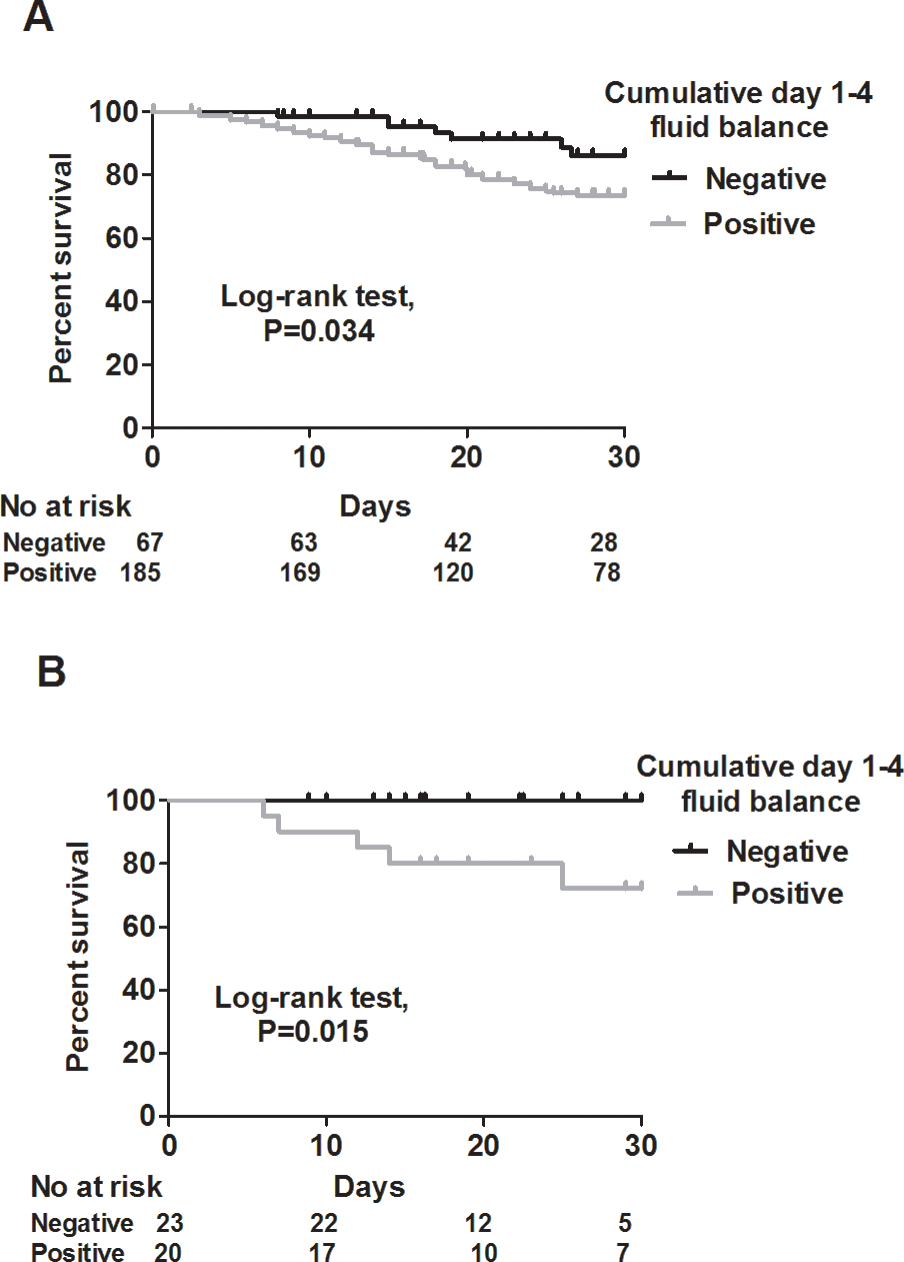
**Kaplan-Meier survival curves for positive (gray) and negative (black) cumulative day 1–4 fluid balance in the ARDS (A) and non-ARDS (B) groups.** ARDS: Acute Respiratory Distress Syndrome.

We used Kaplan-Meier analysis to test the correlation between cumulative day 1–4 fluid balance and 30-day mortality, and found that a negative cumulative day 1–4 fluid balance was associated with a lower risk of mortality (log-rank test, *P =* 0.003) (Fig 2). Given that shock status may confound the correlation between fluid balance and mortality, we then stratified the patients into shock and non-shock groups. In the shock group, even though a negative cumulative day 1–4 fluid status tended to be associated with a lower mortality rate, the statistical power did not reach significance (log-rank test, *P =* 0.396) (Fig 3A). However, in the non-shock group, a negative cumulative day 1–4 fluid balance remained highly correlated with a lower risk of mortality (log-rank test, *P =* 0.008) (Fig 3B). In addition, as the enrolled patients in this study mostly had influenza-related ARDS, and as a negative fluid balance has been recently reported to be associated with a lower risk of mortality in patients with ARDS [14], we further classified the patients into ARDS and non-ARDS groups. Of note, we found a positive correlation between a negative cumulative day 1–4 fluid balance and a lower risk of 30-day mortality in both the ARDS (log-rank test, *P =* 0.034), and non-ARDS (log rank test, *P =* 0.015) groups (Fig 4A and 4B). We then investigated which independent variables could predict 30-day mortality in these 296 patients. Given that the APACHE II score is calculated from vital signs, white blood cell count, hematocrit, platelet count, serum creatinine level, and oxygenation status, we used APACHE II scores to represent severity in the multivariate regression model. In a multivariate Cox proportional hazard regression model adjusted for age, sex, cerebrovascular disease, PaO2/FiO2 ratio and the use of vasopressor, a high APACHE II score (adjusted hazard ratio (aHR) 1.039, 95% confidence interval (CI) 1.001–1.079), the use of ECMO (aHR 2.075, 95% CI 1.088–3.958), and cumulative day 1–4 fluid balance (aHR 1.0880, 95% CI 1.007–1.174) were independently associated with 30-day mortality (Table 3). These findings showed that a negative cumulative day 1–4 fluid balance played a critical role in the patients admitted to the ICU for complicated influenza.

**Table 3:** Cox proportional hazard regression analysis for 30-day mortality.

Characteristics	Univariate		Multivariate	
	HR (95% C.I.)	*P* value	HR (95% C.I.)	*P* value
Age, per 1 year increment	1.011 (0.994–1.029)	0.22	1.011 (0.989–1.035)	0.32
Sex				
Female	1 [Reference]		1 [Reference]	
Male	1.071 (0.614–1.868)	0.81	0.900 (0.496–1.635)	0.73
Cerebrovascular disease				
No	1 [Reference]		1 [Reference]	
Yes	2.29 (1.12–4.679)	0.02	2.115 (0.916–4.885)	0.08
APACHE II, per 1 increment	1.064 (1.031–1.099)	<0.01	1.029 (0.990–1.071)	0.15
P/F ratio, per 1 increment	0.996 (0.992–0.999)	0.03	0.996 (0.992–1.000)	0.07
Hemodialysis				
No	1 [Reference]		1 [Reference]	
Yes	2.224 (1.255–3.941)	<0.01	1.519 (0.721–2.670)	0.24
Use of vasopressors				
No	1 [Reference]		1 [Reference]	
Yes	2.247 (1.268–3.981)	0.01	1.388 (0.721–2.670)	0.33
Cumulative day 1–4 fluid balance, per 1 liter increment	1.169 (1.088–1.256)	<0.01	1.095 (1.012–1.186)	0.03

HR: hazard ratio; C.I.: confidence interval; APACHE: acute physiology and chronic health evaluation; P/F ratio: PaO2/FiO2 ratio.

## Discussion

In this study we investigated the cumulative fluid status in influenza-related critically ill patients. We found that these patients had a relatively modest fluid requirement, and that a negative day 4 cumulative fluid balance was associated with a lower risk of 30-day mortality. These findings suggest the crucial role of fluid balance in the management of patients with complicated influenza, particularly in those who are critically ill without shock.

Fluid resuscitation is the cornerstone of the early management of patients with sepsis [2], and timely appropriate fluid administration has been shown to modulate inflammatory responses [15]. Three high-quality, large-scale trials have reported on initial resuscitation in septic shock [16–18], however the fluid management strategy following hemodynamic stabilization remains unclear. Hjortrup *et al*. recently reported that a protocol restricting resuscitation fluid in adults with septic shock after initial management on day 1 reduced the volume of resuscitation fluid by approximately 1200 mL on day 5, although there was no significant effect on mortality [19]. These results are consistent with the findings in our patients with shock (Fig 3A) and highlight the difficulty in clearly separating the net effect of later fluid management from the impact of initial fluid rescue in septic shock. Sepsis has a wide range of pathophysiological and clinical manifestations resulting from a wide range of pathogens and distinct disease status, and therefore fluid administration should be individualized according to specific host-pathogen interactions [9]. Vincent and De Backer proposed four distinct stages of fluid resuscitation including rescue, optimization, stabilization, and de-escalation [20], and this reflects the need for individualized fluid strategies based on the time-course and host-pathogen interactions in sepsis [21]. However, it can be difficult to delineate each stage in diverse clinical conditions due to complex underlying pathophysiological interactions. For example, inadequate initial fluid rescue may distort the subsequent physiological responses. Another crucial issue in delineating the stage is that severe endothelial injury in patients with sepsis can lead to a non-fluid responsive status [7], and fluid non-responsiveness may lead to continuous fluid administration due to the lack of parameters to monitor endothelial function.

The role of cumulative fluid balance in sepsis is not fully understood. Cunha *et al*. investigated cumulative fluid balance after weaning from vasopressors in patients with septic shock, and they found that cumulative fluid balance tended to continue to increase even after recovery from shock [22]. Thus, despite the increasing evidence regarding the potential detrimental impacts of fluid overload, there is a need to clarify the impact of fluid balance and the optimal fluid administration following hemodynamic stabilization. In this study, we enrolled patients with virology-proven influenza, characterized by the relative modest requirement of fluid rescue, and this approach contributes to minimizing the potential confounding effect of inadequate early fluid rescue, as we showed that a negative fluid balance was highly correlated with a lower risk of mortality in the patients with influenza without shock (Fig 3B). In addition, the serial fluid balance in the survivor group as demonstrated by a positive fluid balance on day 1 and day 2, a less positive fluid balance on day 3, and a gradually negative fluid balance between day 4 and day 7, may reflect the rescue, optimization-stabilization, and de-escalation stages of fluid administration in critically ill patients with influenza (Table 2).

Our finding of benefits in reducing the risk of mortality in the patients with ARDS with a negative day 4 cumulative fluid status is consistent with the crucial role of a conservative fluid strategy in ARDS (Fig 4A). The Fluids and Central Catheters Trial (FACCT) investigated the causal effect of a positive fluid balance on outcomes of patients with acute lung injury, and found that the patients who received conservative fluid therapy had a shorter duration of ventilation and ICU stay than those with liberal fluid therapy [23]. Furthermore, Semler *et al*. recently reported subgroup analysis of the FACCT with patients having a low initial central venous pressure (0–8 mmHg), and reported that the patients who received conservative fluid therapy had a lower mortality rate (17% vs. 36%, *P =* 0.005) than those with liberal fluid therapy [14]. Notably, they found that the patients who received liberal fluid therapy had a positive cumulative fluid balance (7100±9200 mL) on day 7, whereas a balanced cumulated fluid status (0±8700 mL) was found in the patients who received conservative fluid therapy. Similarly, Rosenberg *et al*. reviewed the fluid balance in the ARDSnet cohort and found that a negative cumulative fluid balance on day 4 was associated with a lower risk of mortality [24]. These data are consistent with our findings of a cumulative positive fluid balance (4474±5313 mL) on day 7 in the non-survivors compared to a balanced fluid status (510±4429 mL) in the survivors (Table 2). Collectively, a conservative fluid strategy appears to be critical in the management of ARDS, including influenza-related ARDS, and future studies would be interested in exploring types of fluids and the role of blood products.

We also found that a negative cumulative fluid balance on day 4 was correlated with a lower risk of mortality in both the patients with and without ARDS (Fig 4A and 4B). These findings indicate that a balanced fluid status may have systemic benefits, given that fluid overload leads to a systemically increased capillary transmural hydrostatic pressure. Genga and Russell recently summarized the current evidence regarding the association between a positive fluid balance and a worse outcome, and suggested that systemic tissue edema and endothelial dysfunction are key links between fluid overload and a poor outcome [25]. For example, increased pulmonary edema in ARDS impairs oxygenation and increases the work of breathing [14], and cerebral edema impairs cerebral blood flow autoregulation and reduces the level of consciousness with an elevated risk of nosocomial infection and overall mortality [26, 27]. In addition, the endothelium has been found to both provide a barrier and also to play an active role in modulating vascular tone and vascular permeability [7, 28], and therefore endothelial dysfunction may lead to poor fluid responsiveness in patients with sepsis. Moreover, fluid overload in patients with endothelial dysfunction can exacerbate high capillary transmural hydrostatic pressure and vascular leak. A number of biomarkers for endothelial damage have been identified, including glycocalyx [29], soluble thrombomodulin [30], and antiopoietin-2 [31], and these biomarkers may also serve as adjunctive therapeutic targets with the aim of restoring endothelial function and avoiding fluid overload in patients with sepsis.

Notably, a high mortality rate (18.6%, 55/296) was found in the reported influenza epidemic, and such a high mortality rate may be attributed by the high disease-severity of enrolled subjects as evidenced by the high proportion of severe ARDS (49.0%, 145/296). Indeed, managing such an abrupt increase of critically ill influenza patients during the influenza epidemic is challenging for the healthcare system. However, preparedness for influenza epidemic is a complex issue and requires the tight cross-department cooperation, including vaccination strategy, early administration of antiviral medications, hygiene interventions, and the standardized medical care [32, 33]. Currently, the TSIRC is working on proposing practical suggestions, including fluid strategy, of caring patients with influenza to a panel, which is drafting Taiwanese clinical practice guidelines for pneumonia; therefore, the findings of this study might be translated into clinical practice in the future.

There are several limitations to this. First, this is a retrospective study, and 11.9% (40/336) of the patients did not have complete early fluid data. However, the mortality rate was similar (20.0% (8/40) vs. 18.6% (55/296)) between the included patients and those excluded due to incomplete initial input-output data. Second, data on the dosage of vasopressors were lacking, however data on the use and duration were available, which allowed us to define the patient with shock. Third, detailed data on fluid administration and de-escalating protocols were not available. However, there is currently no consensus on the optimal fluid management in patients with complicated influenza. In addition, the eight participating hospitals in this study are teaching hospitals with qualified ICU intensivists whose exclusive duty is to care for ICU patients; therefore, the fluid management should have been based on the condition and response of the patients. Fourth, data on the subtype of influence were not available for all of the patients. In real-world practice, antiviral agents will be prescribed immediately in patients with positive RIDT, and thus there may be a delay in sending samples to the CDC for RT-PCR testing.

## Conclusions

In conclusion, fluid balance plays a critical role in the management of patients with sepsis, and our data showed an association between a negative cumulative day 1–4 fluid balance and a lower risk of 30-day mortality in our critically ill patients with influenza, particularly in those without shock. This study may provide information on the optimal fluid strategy in specific septic conditions such as complicated influenza. Further studies are warranted to investigate fluid balance in other septic conditions and to identify prognostic and therapeutic molecular targets to guide and improve fluid management in patients with sepsis.

## Ethics approval

Taichung Veterans General Hospital CE16093A, National Taiwan University Hospital 201605036RIND, Taipei Veterans General Hospital 2016-05-020CC, Tri-Service General Hospital 1-105-05-086, Chang-Gung Memorial Hospital 201600988B0, China Medical University Hospital 105-REC2-053(FR), Kaohsiung Medical University Hospital KUMHIRB-E(I)-20170097, Kaohsiung Chang-Gung Memorial Hospital 201600988B0

## Supporting information

S1 Dataset (XLSX)

## Abbreviations

aHR: adjusted hazard ratio
APACHE II: Acute Physiology and Chronic Health Evaluation II
ARDS: acute respiratory distress syndrome
CDC: Centers for Disease Control
ECMO: extracorporeal membrane oxygenation
FACCT: Fluids and Central Catheters Trial
ICU: intensive care unit
RIDT: rapid influenza diagnostic test
TSIRC: Taiwan Severe Influenza Research Consortium
